# A Novel Antibody against Human Properdin Inhibits the Alternative Complement System and Specifically Detects Properdin from Blood Samples

**DOI:** 10.1371/journal.pone.0096371

**Published:** 2014-05-05

**Authors:** Diana Pauly, Benedikt M. Nagel, Jörg Reinders, Tobias Killian, Matthias Wulf, Susanne Ackermann, Boris Ehrenstein, Peter F. Zipfel, Christine Skerka, Bernhard H. F. Weber

**Affiliations:** 1 Institute of Human Genetics, University of Regensburg, Regensburg, Germany; 2 Institute of Functional Genomics, University of Regensburg, Regensburg, Germany; 3 Department of Infection Biology, Leibniz Institute for Natural Product Research and Infection Biology, Jena, Germany; 4 Klinik und Poliklinik für Rheumatologie und Klinische Immunologie, Asklepios Klinikum Bad Abbach, Bad Abbach, Germany; 5 Department of Infection Biology, Friedrich Schiller University, Jena, Germany; University of Leicester, United Kingdom

## Abstract

The complement system is an essential part of the innate immune system by acting as a first line of defense which is stabilized by properdin, the sole known positive regulator of the alternative complement pathway. Dysregulation of complement can promote a diversity of human inflammatory diseases which are treated by complement inhibitors. Here, we generated a novel blocking monoclonal antibody (mAb) against properdin and devised a new diagnostic assay for this important complement regulator. Mouse mAb 1340 specifically detected native properdin from human samples with high avidity. MAb 1340 inhibited specifically the alternative complement mediated cell lysis within a concentration range of 1–10 µg/mL. Thus, *in vitro* anti-properdin mAb 1340 was up to fifteen times more efficient in blocking the complement system as compared to anti-C5 or anti-Ba antibodies. Computer-assisted modelling suggested a three-dimensional binding epitope in a properdin-C3(H_2_O)-clusterin complex to be responsible for the inhibition. Recovery of properdin in a newly established sandwich ELISA using mAb 1340 was determined at 80–125% for blood sample dilutions above 1∶50. Reproducibility assays showed a variation below 25% at dilutions less than 1∶1,000. Systemic properdin concentrations of healthy controls and patients with age-related macular degeneration or rheumatic diseases were all in the range of 13–30 µg/mL and did not reveal significant differences. These initial results encourage further investigation into the functional role of properdin in the development, progression and treatment of diseases related to the alternative complement pathway. Thus, mAb 1340 represents a potent properdin inhibitor suitable for further research to understand the exact mechanisms how properdin activates the complement C3-convertase and to determine quantitative levels of properdin in biological samples.

## Introduction

The complement system serves as a bridge between the innate and the adaptive immune system. More than 40 blood proteins interact in cascades to eliminate blood and tissue infectious agents by opsonization, anaphylatoxins and cell lysis. However, activated complement is a double-edged sword, capable of protecting from pathogens as well as causing self-tissue damage. Complement dysregulation is caused by mutations in complement genes, the presence of autoantibodies or large tissue damage [1], [2]. There is abundant evidence for complement activation in several autoimmune, eye and kidney diseases [1], [3].

Disease-associated variants in several complement genes in patients with age-related macular degeneration (AMD) or atypical haemolytic-uraemic syndrome (aHUS) directed the attention of therapeutic interventions to the alternative complement pathway [4]–[6]. Spontaneous or surface-dependent hydrolysis of complement protein 3 (C3) to C3(H2O) specifically activates this pathway in human blood. C3(H2O) binds complement factor B and interacts with complement factor D to form a fluid phase C3-convertase which cleaves C3 in C3a and C3b. C3b opsonizes target surfaces and binds factor B, which is cleaved by factor D, yielding Bb. Properdin stabilizes five to ten-fold both the fluid-phase (C3(H2O)Bb) and surface-bound (C3bBb) C3-convertase of the alternative pathway [7]. Complement factor H (CFH) dissociates the C3-convertase and in combination with factor I inactivates the C3b protein [7], [8]. In a positive feedback loop, C3-convertase cleaves C3 and an additional C3b molecule complements C3bBb to form the C5-convertase. C5 cleavage initiates the terminal complement pathway and mediates inflammation as well as the formation of a cell membrane attack [9].

Properdin, the only known positive regulator of the complement system, escalates and initiates the alternative pathway [7], [10]. It is expressed in various cell types, mainly leukocytes, resulting in a systemic serum concentration of 4–25 µg/mL [11], [12]. The glycoprotein with a molecular weight of about 50 kDa consists of seven thrombospondin type I repeats (TSR) [13], [14]. Under physiological conditions, humoral properdin exists as cyclic dimers, trimers and tetramers in a fixed ratio of 26∶54∶20 (dimer:trimer:tetramer) [15]. Recently, structural studies of properdin multimers showed four TSR subunits of two monomers forming a vertex which interacts with the C3-convertases [16]. In this complex, properdin TSR 4 and 5 interact with the C3α-chain while the Ba and Bb subunits of factor B bind to properdin [16], [17]. Which properdin subunit interacts with Ba or Bb remains unknown [16]–[19]. Stabilization of the convertases is opposed by CFH-mediated dissociation of Bb although direct inhibitory regulation of properdin by CFH is not known.

Mutations in negative regulators of the C3-convertase result in pathogenic tissue damage [20]–[24] and several studies in mice implicate properdin in the pathogenesis of complement-mediated tissue injury [25]. Miwa et al. described an alternative pathway-dependent pathology in a renal ischemia-reperfusion mouse model which was significantly ameliorated by properdin depletion [26]. A similar effect was reported in a mouse model for abdominal aortic aneurysm, where aneurysm formation was controlled by autoantibodies and properdin activity [27]. Additionally, properdin-deficient mice also showed a reduced severity of tissue damage in two different models of arthritis [28], [29]. Antibody-mediated inhibition of properdin in these animal models prevented or ameliorated disease development. Disease associated complement activation in humans showed complement consumption and can results in decreased properdin concentrations [30]. Consistent with the animal models, rheumatoid arthritis patients showed a decreased properdin concentration in synovial fluid [31]. Autoantibodies and properdin are also involved in pathologies of neuromyelitis optica, Schönlein-Henoch syndrome and systemic lupus erythematosus (SLE) where systemic properdin concentrations are also decreased [32]–[34]. These studies indicate the potential of properdin as a therapeutic target in complement-mediated disorders.

Several complement antibody-therapeutics are currently under development, but only Eculizumab (anti-C5) is approved to treat alternative pathway-associated disorders, aHUS and paroxysmal nocturnal hemoglobinuria (PNH) [35]. Clinical evaluation of Eculizumab demonstrated an improvement for aHUS patients and a favorable tolerability profile [36].

Regulators of complement activation can prevent or facilitate formation of convertases. Supplements with soluble negative complement regulators are in clinical phase I (complement receptor 1) or preclinical studies (CFH constructs, protectin) [35]. For the inhibition of properdin, a monoclonal anti-properdin antibody was described in 2000 but no further preclinical or clinical studies have been pursued [37]. The main concern in the context of complement inhibition is the risk of increased susceptibility to infection. However, if properdin is the target, this issue can be circumvented with prophylactic vaccination against *Neisseria meningitides*, the only known life-threatening infection in properdin-deficient patients [38].

In this study, we generated a novel mouse anti-properdin antibody and characterized its specificity and avidity to human properdin. We demonstrated that our monoclonal antibody inhibits complement activation and allows defining properdin serum levels in patients with complement-mediated diseases. Thus, mAb 1340 may provide a promising future complement therapeutic and analytical tool.

## Materials and Methods

### Patient samples and ethics statement

Human blood samples were taken after approval of the local ethics committees (University Regensburg, references 12-101-0241 and 12-101-0074). All donors signed a written consent form. Patients with age-related macular degeneration (AMD, n = 20) were recruited at the University Regensburg. Patients with systemic lupus erythematosus (SLE, n = 6), connective tissue diseases (CTD, n = 10), polymyalgia rheumatica (PR, n = 31), rheumatoid arthritis (RA, n = 38), spondyloarthritis (SPA, n = 40) and systemic sclerosis (SSc, n = 16) were recruited at the Asklepios Klinikum Bad Abbach. All samples of patients with rheumatic diseases were obtained prior to initiation of corticosteroid or immunosuppressive therapy. Healthy blood donors (controls, n = 26) were randomly chosen students and staff members of the University Regensburg.

All mice were handled according to the good animal practice in science. This study was carried out in strict accordance with the recommendations in the Guidelines for the Care and Use of Laboratory Animals of the Federation of European Laboratory Animal Science Associations. The protocol was approved by the Committee on the Ethics of Animal Experiments of the regional agency for animal health Regierung der Oberpfalz, Veterinärwesen (permit number: TV 54-2532.4-04/12). All immunizations and bleedings were performed under inhalational anesthesia (Isoflurane), and all efforts were made to minimize suffering.

### Materials

Monoclonal anti-properdin antibodies (A233, A235), anti-C5 antibody (A217) and Anti-Ba (A225) were obtained from Quidel, San Diego, USA. MAb anti-botulinum toxin (BoNT) was generated previously [39]. Cell culture reagents were purchased from Life technologies, Darmstadt, Germany. All chemicals without separate reference were commercially available from Sigma-Aldrich, Munich, Germany.

### Generation of monoclonal antibodies

Mouse monoclonal antibodies against human properdin were generated as previously described [39]. Briefly, six weeks-old female Balb/c mice (Charles River Laboratories, Sulzfeld, Germany) were immunized five times with 25 µg human, purified properdin (Quidel, San Diego, USA). Antigens were applied subcutaneously in complete Freund's adjuvants for priming immunization and incomplete Freund's adjuvants for follow-up immunizations. Spleen cells were isolated and fused with myeloma cell line P3-X63-AG8.653 (American Type Culture Collection, Manassas, USA) using a ratio of 2∶1 with polyethylene glycol 1500 (Roche Diagnostics, Mannheim, Germany) according to standard procedures [40]. Cells were cultivated on BALB/c thymocytes as feeder cells in RPMI 1640 media, containing 20% fetal calf serum, 50 µM 2-mercaptoethanol, 50 U/mL recombinant murine interleukin 6, 1% glutamine, 5.8 µM azaserine and 100 µM hypoxanthine. On day 10–20 post fusion, hybridoma supernatant was tested for properdin-binding in an indirect enzyme-linked immunoassay (ELISA). Positive hybridomas were isolated and subcloned by limiting dilution. Stability and clonality of hybridomas were tested by intracellular immunoglobulin staining and flow cytometry analysis [40]. Monoclonal antibodies were purified using HiTrap protein G HP affinity column (GE Healthcare Life Science, Piscatawa, USA). Purity was checked by SDS-PAGE. The concentration of purified antibodies was measured using a NanoDrop 1,000 spectrometer. Isotype was determined using a mouse isotyping kit (AbD serotec, Kidlington, UK). Antibody biotinylation was performed as described before [39].

### Indirect ELISA for antibody binding

MaxiSorp plates (Nalgene Nunc, Penfield, USA) were coated with 0.5–1 µg/mL antigen in PBS (overnight, 4°C). All incubation steps were completed with three subsequent washing steps with wash buffer (PBS, 0.1% Tween 20). After blocking with blocking buffer (PBS, 0.1% Tween 20, 2% skimmed milk) antibodies serially diluted in blocking buffer (1,000–0.1 ng/mL) were added (1 h). Detection was performed with a peroxidase-conjugated anti-mouse immunoglobulin G (IgG) antibody (Jackson ImmunoResearch Laboratories, West Groove, USA) and 3,3′,5,5′-tetramethylbenzidine (TMB, Seramun, Wolzig, Germany). Signal was determined at 450 nm.

### cDNA cloning of mouse properdin

Total RNA was extracted from mouse liver (RNeasy MiniKit, QIAGEN, Hamburg, Germany). Full-length murine properdin (NM_008823.3, nt 71–1462) was amplified with oligonucleotide primers 5′-GAATTCATGCCTGCTGAAATGCAAGCC-3‘ and 5‘-CTCGAGGGGTTTCTTCTCTTCTGGGTCT-3‘. The PCR product was cloned into expression vector pEXPR-IBA103 (IBA, Goettingen, Germany). Human embryonic kidney cells 293-EBNA (HEK, Life Technologies, Carlsbad, CA, USA) were transfected with TransIT-LT1 Transfection Reagent (Mirus, Madison, WI, USA). The supernatant was purified using Gravity flow Strep-Tactin Sepharose Column (IBA, Goettingen, Germany). Expression and purity was confirmed by Western blot (antibody: StrepMAB-Classic conjugated to horseradish peroxidase, IBA, Goettingen, Germany) and SDS-PAGE.

### Immunoprecipitaton of properdin

Tosylactivated dynabeads (Life Technologies, Carlsbad, USA, 5 mg) were conjugated to 100 µg mAb 1340 according to the manufacture's protocol. Pooled human Serum (NHS, 1.5 mL) was incubated with mAb 1340-dynabeads (50 µL, 1 h). After washing with wash buffer, proteins were eluted using non-reducing Laemmli sample buffer and denaturation at 95°C (10 min) [41].

### Protein gel analysis and Western blot analysis

After immunoprecipitation, eluted samples and purified proteins were denatured (5–10 min, 95°C) and analyzed on a non-reducing 6–12% SDS-PAGE with subsequent Coomassie staining (detection limit 1 µg) as described before [41]. Proteins were separated and transferred on a polyvinylidene difluoride membrane applying the semi-dry blotting method [42]. After blocking with blocking buffer (PBS, 0.1% Tween 20, 2% skimmed milk), the membrane was incubated with 1 µg/mL mAb 1340 (5 mL in blocking buffer, 1 h). The washed membrane was treated with a peroxidase conjugated anti-mouse IgG antibody (in blocking buffer, 30 min). After six washing steps with PBS, antibody binding was detected using ECL solution (100 mM Tris/HCl pH 8.8, 1.3 mM luminol, 0.6 mM p-coumaric acid, 4.6 mM hydrogenperoxide) (detection limit 10 ng).

### LC-MS/MS analysis

Visible bands from Coomassie-stained gels were excised, washed and tryptically digested as published previously [43]. Resulting peptides were used for nano-LC-MS/MS-analysis on a TripleTOF 5600+ QTOF mass spectrometer equipped with an Ultimate 3000 nano-HPLC with precolumn concentration (precolumn: 100 µm I.D., 2 cm, Acclaim PepMap100, 5 µm (Dionex, Idstein, Germany); HPLC column: 75 µm I.D., Acclaim PepMap100, 3 µm; 300 nL/min (Dionex, Idstein, Germany)). Samples were separated using a 30 min binary gradient from 4–30% B (solvent A: 0.1% formic acid; solvent B: 0.1% formic acid in acetonitrile). The mass spectrometer repeatedly acquired a survey scan and MS/MS spectra of the four most intensive ions for 0.2 s each. Database searches were accomplished using the Mascot algorithm (version 2.3) on the Uniprot-database (version 05/2013).

### Lysis assays

Complement activity of the alternative pathway was measured in a hemolysis assay with sheep erythrocytes [44], [45]. We intended to avoid false-negative results for antibody testing and used erythrocytes which were more resistant to lysis by human serum [46]. Antibodies were serially diluted in MgEGTA buffer (20 mM HEPES, 144 mM NaCl, 10 mM EGTA, 7 mM MgCl_2,_ pH 7.4) and preincubated with 20% NHS in MgEGTA buffer (15 min, 37°C). Sheep red blood cells (1×10^8^) were treated with 20% NHS-antibody mixtures in MgEGTA buffer for 30 min (37°C). Lysis was determined at 414 nm.

Alternatively, blocking activity of mAb was tested in an *Escherichia coli* (*E. coli*) lysis assay. MAb in different concentrations were preincubated either with 40% NHS in MgEGTA buffer (alternative pathway) or 20% NHS in PBS (classical pathway) (30 min, 37°C). *E. coli Stbl3* (Life Technologies, Carlsbad, CA, USA) cultures with an optical density of 1 at 600 nm were added. *E. coli* cells were incubated at 37°C and growth was determined after 3 h at 600 nm.

### Determination of antibody sequence

The cDNA of hybridoma cell line 1340 was synthetized using the RNeasy Plus Mini Kit (Qiagen, Hamburg, Germany) and the Cloned AMV First-Strand cDNA Synthesis Kit (Life technologies, Darmstadt, Germany). In order to amplify the IgG variable region of heavy and light chain, the Mouse IgG Library Primer set (Progen, Heidelberg, Germany) was used according to the manufacturer's protocol. The purified PCR products were ligated into a pGEM-T plasmid (Promega, Mannheim, Germany). After heat shock-induced plasmid transfection into competent *E. coli DH5α* (Life Technologies, Carlsbad, CA, USA) the plasmid was amplified in an overnight culture and isolated using the NucleoSpin Plasmid kit (Macherey-Nagel, Düren, Germany). For Sanger sequencing a reaction mix with M13 primer (Promega, Mannheim, Germany) and the BigDye Terminator v3.1 cycle sequencing kit (Life Technologies, Carlsbad, USA) were used.

### Depletion of properdin from human blood sample

MAb 1340 was coupled to a HiTrap NHS-activated affinity column (GE Healthcare Life Science, Piscatawa, USA) according to the manufacturer's instructions. Properdin was depleted from human serum and plasma by recirculation 3 ml over the mAb 1340 column for 2 h. Through flow was tested in the reported sandwich ELISA and Western blot for properdin concentration. There was no properdin detectable.

### Sandwich ELISA for properdin detection

For development and optimization of the properdin specific sandwich ELISA we tested different antibodies (mAb 1340, mAb 149 (inhouse anti-properdin mAb), mAb A233, mAb A235, pAb goat anti-properdin (Complement technologies, Tyler, USA)), buffer (PBS, PBS/T, 1% BSA/PBS/T, Casein buffer, 2% skim milk in PBS/T, MgEGTA buffer) and detection systems (streptavidin-peroxidase and poly-horseradish peroxidase). The studies with human blood were done with the most reliable, sensitive and practical assay of the pre-evaluation studies.

MaxiSorp plates were coated with 2 µg/mL mAb A235 in PBS overnight at 4°C. After blocking with blocking buffer (1 h), plates were washed and incubated with different samples (1 h): serial dilutions of pooled human plasma (NHP) or NHS in PBS (1∶10–1∶156,250), properdin (100 ng/mL) spiked into properdin-depleted NHS or NHP, different species sera (1∶1,250), mouse properdin (500 ng/mL), bovine serum albumin (500 ng/mL) and unknown serum samples (1∶700) in PBS. Subsequently, washing and incubation with biotinylated mAb 1340 was performed (1 h). SA-POD (Jackson ImmunoResearch Laboratories, West Groove, USA) and TMB were used for signal development. Optical density was measured at 450 nm. Six different NHP or NHS standard curves on one day or on different days were performed for intra-assay and inter-assay variation, respectively. Coefficient of variation (CV) was calculated by means and standard deviations of absorbance values at 450 nm.

### Software

The structure of the variable region of mAb 1340 was modeled on the VBASE2 server [47], the Rosetta online server [48] and with the proABC method [49]. Complementarity-determining regions (CDR) prediction was performed with abYsis, VBASE2, Rosetta and IMGT methods [50]. Visualizations of structures were done with UCSF Chimera [51]. Data analyses were performed with GraphPad Prism (GraphPad Software, San Diego, USA).

## Results

### Newly generated monoclonal anti-properdin antibody binds human properdin with high avidity

**Figure 1:**
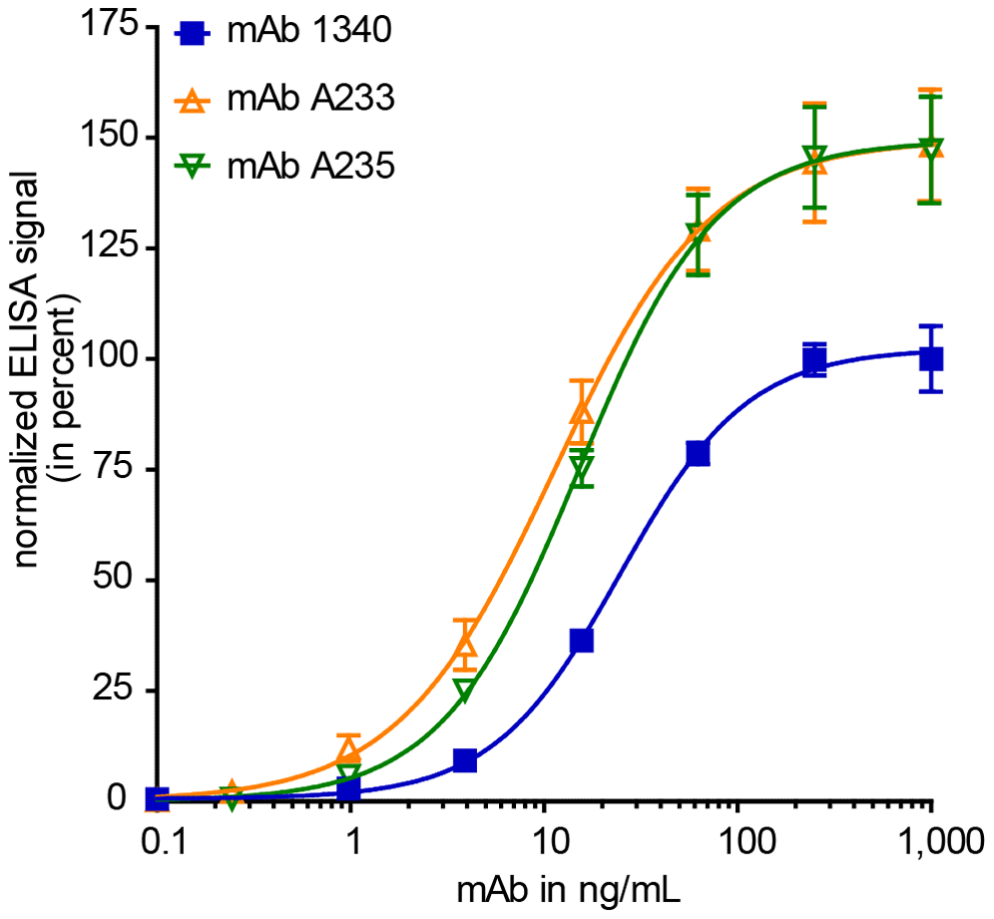
Anti-properdin antibodies show high avidity towards human properdin. The binding strengths of newly generated mAb 1340 as well as commercially available mAb A233 and mAb A235 were tested in an indirect ELISA. MAbs were serially diluted (1,000–0.1 ng/mL) and properdin binding was detected with a peroxidase conjugated anti-mouse IgG antibody. MAb 1340 (EC50 25 ng/mL), mAb A233 (EC50 11 ng/mL) and mAb A235 (EC50 15 ng/mL) showed comparable binding strengths towards immobilized human properdin. Shown are means (n = 9±s.e.m.) out of three independent experiments each with three replicates. After background subtraction, data were normalized to 1,000 ng/mL mAb 1340 reactivity against properdin (set to 100%).

Mice were immunized with purified human properdin for the generation of specific and high-affinity antibodies. The selected mouse showed a specific serum titer of 1∶220,000 against the target protein. Among 1,900 screened hybridoma clones, a stable cell line #1340 secreted anti-properdin antibodies. The binding strengths of different monoclonal antibodies were tested in an ELISA with immobilized human properdin (**Figure 1**). MAb 1340, together with two commercial anti-properdin mAb, i.e. mAb A233 and A235 interacted concentration-dependent with the designated antigen. We detected comparable avidities for mAb 1340, A233 and A235 with half maximal effective concentrations (EC50) of 0.16 nM, 0.07 nM and 0.1 nM, respectively. The described antibodies showed similar binding strengths as an established anti-complement mAb Eculizumab (anti-C5 mAb, 0.12 nM) and even a higher mAb-antigen interaction as the routinely used therapeutic mAb Bevacizumab (anti-VEGF, 0.8 nM) [52], [53]. In consideration of a broad affinity range from 1 pM to 1 nM of therapeutic antibodies the newly described mAb 1340 displays a high avidity towards properdin [54]. The binding strength is important for later *in vivo* potency as well as future doses and dosing intervals.

### MAb 1340 interacts with native human properdin multimers

**Figure 2:**
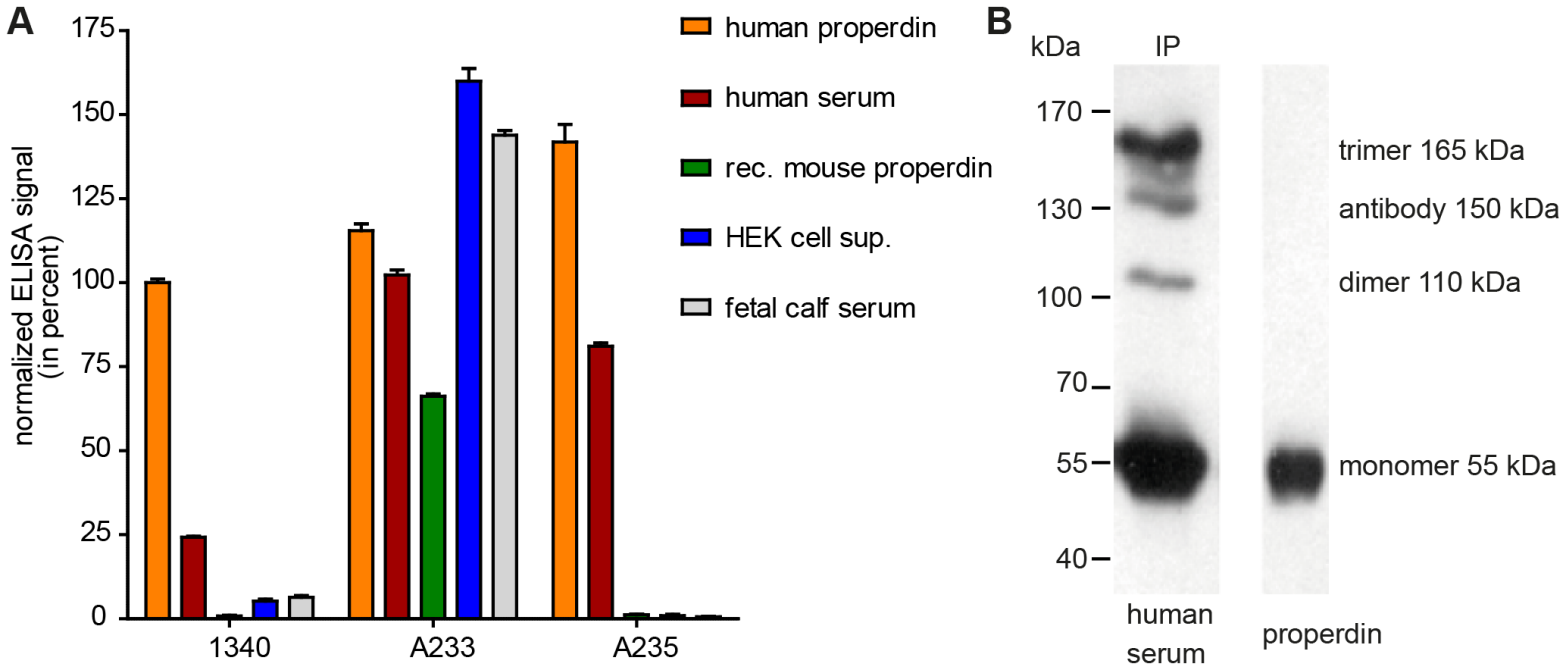
Monoclonal antibodies specifically detect properdin in human serum. (**A**) Purified human properdin, human serum, recombinant mouse properdin, human embryonic kidney (HEK) cell supernatant and fetal calf serum were immobilized on an ELISA plate. In house mAb 1340 (1 µg/mL) and commercial mAb A235 (1 µg/mL) detected only purified human properdin or human serum, respectively. MAb A233 (1 µg/mL) detected all antigens tested. Shown are the respective means (± s.e.m.) for two independent experiments. After background subtraction data were normalized to mAb 1340 reactivity against properdin (set to 100%). (**B**) Human properdin was isolated from human serum by immunoprecipitation (IP) using mAb 1340 (left lane). The precipitated proteins and purified control properdin (right lane) were separated by non-reducing, denatured SDS-PAGE. Western blot detection was performed with mAb 1340 and a peroxidase conjugated anti-mouse IgG antibody. The generated mAb precipitated and detected properdin monomer (∼55 kDa), dimer (∼110 kDa) and trimer (∼165 kDa), respectively.

MAbs are also characterized by a unique specificity, which was tested by ELISA for anti-properdin mAbs. MAb 1340 and commercial A235 specifically recognized human purified properdin or properdin in human serum (**Figure 2A**). Therefore, mAb A235 was used as a control antibody in all further experiments. Heat denatured or reduced properdin was not detected using mAb 1340 (data not shown). The novel mAb did not react with unspecific proteins like mouse properdin, supernatant derived from human embryonic kidney cells or fetal calf serum (**Figure 2A**). In contrast, a widely used commercial mAb A233 detected all immobilized antigens and did not discriminate between human, mouse and fetal calf properdin as well as cell supernatant without properdin above 1 µg/mL but not at lower concentrations. A233 cross-reactivity could be due to an enhanced non-specific attraction between proteins and not necessarily to antigen-antibody binding.

These results indicated a high specificity of mAb 1340 for properdin and initiated a deeper characterization of the mAb 1340 interaction partners.

Properdin interacts in a multimeric structure with several other human serum components. We used mAb 1340 to immunoprecipitate properdin multimers and associated proteins from serum samples. The antibody detected different aggregated properdin oligomers in a Western blot analysis (**Figure 2B**). The ratio of the densitometric intensity of properdin trimer (165 kDa): dimer (110 kDa): monomer (55 kDa) was 7∶1∶18. This ratio differed from the previously described properdin oligomer ratio in blood [15] and maybe due to a non-natural heat treatment-dependent oligomer-aggregation during SDS-PAGE [55].

**Figure 3:**
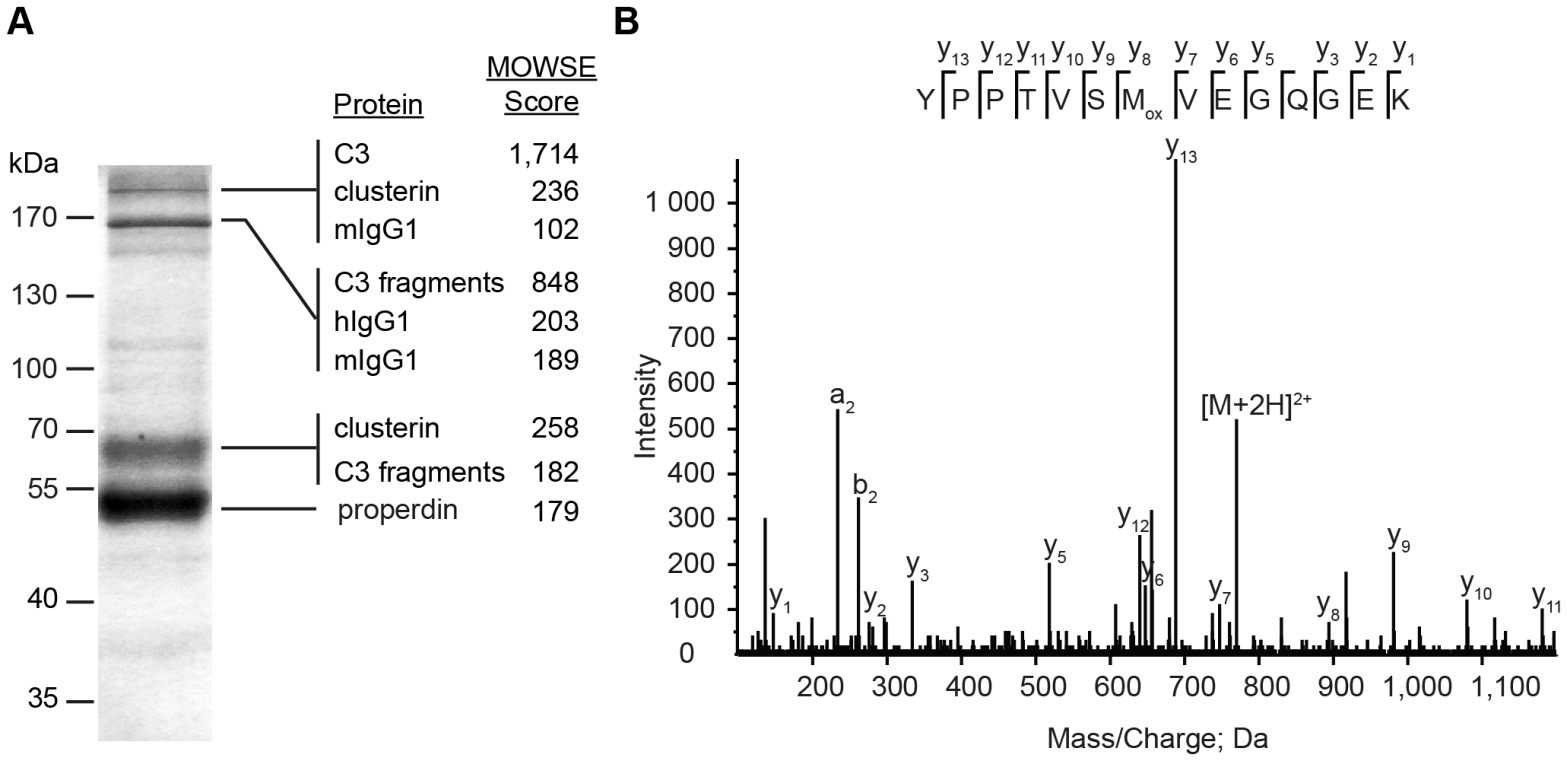
MAb 1340 precipitates properdin, complement factor 3, clusterin and immunoglobulins from human serum. (**A**) Proteins from human serum binding to mAb 1340 were isolated by coimmunoprecipitation. After size separation in an SDS-PAGE, protein fractions were identified using Coomassie staining and combined LC-MS/MS analysis. MAb 1340 precipitates complement factor C3(H_2_O) (>170 kDa) as well as C3-fragments (<170 kDa), clusterin (53 kDa), immunoglobulins (150 kDa, murine IgG (mIgG), human IgG (hIgG)) and properdin (55 kDa). C3 fragments detected at different molecular sizes, included: C3 β chain, C3c alpha chain fragments 1 and 2, C3dg at >170 kDa; C3 β chain, C3c alpha chain fragments 1, C3dg at <170 kDa and C3 β chain at 68 kDa. The confidence index for each identified protein is given by the molecular weight search (MOWSE) score. (**B**) Exemplary MS/MS-spectrum matching the properdin peptide YPPTVSMVEGQGEK (amino acids 409–422) is shown. Detected C-terminal ions (y-ions) are annotated within the peptide sequence.

Native properdin multimers bind the C3-convertases of complement [7]. Using mAb 1340 as a matrix we identified components of a mAb 1340-immunoprecipitated protein mixture at different sizes by mass spectrometry (**Figure 3A**). Properdin was identified at a 55 kDa band. An exemplary mass spectrum for amino acids 409–422 of properdin showed characteristic fragment ions (**Figure 3B**). Additionally, C3(H_2_O) (>170 kDa) and C3 fragments (170 kDa, 68 kDa) were identified. Furthermore, clusterin (53 kDa) and human immunoglobulins (150 kDa) were discovered as binding partners of properdin immobilized on mAb 1340 (**Figure 3A**). Factor Bb, the interaction partner of C3b in the C3-convertase, was not detected. With the use of mAb 1340, we identified novel interaction partners like IgG and clusterin that seemed to include in a multimeric properdin protein complex in soluble human serum.

The identified properdin interaction partners did not bind the properdin epitope of mAb 1340, which was a discontinuous amino acid sequence in the tertiary structure of properdin (**Figure S1**). Three docking algorithms predicted different TSR subunits of a properdin monomer for interaction with mAb 1340 *in silico*. Properdin monomers form multimeric structures with connecting loops, consisting of four TSRs [16]. The putative epitope of mAb 1340 could be located in this crucial interaction point of properdin monomers, which resulted in different docking predictions to the monomer (**Figure S1D**). Binding of mAb 1340 to single TSR subunits showed no result in Western blot analysis or competitive ELISA (**Figure S2**). The only binding partner for mAb 1340 was a structural epitope of full length human properdin.

### MAb 1340 inhibits properdin function

**Figure 4:**
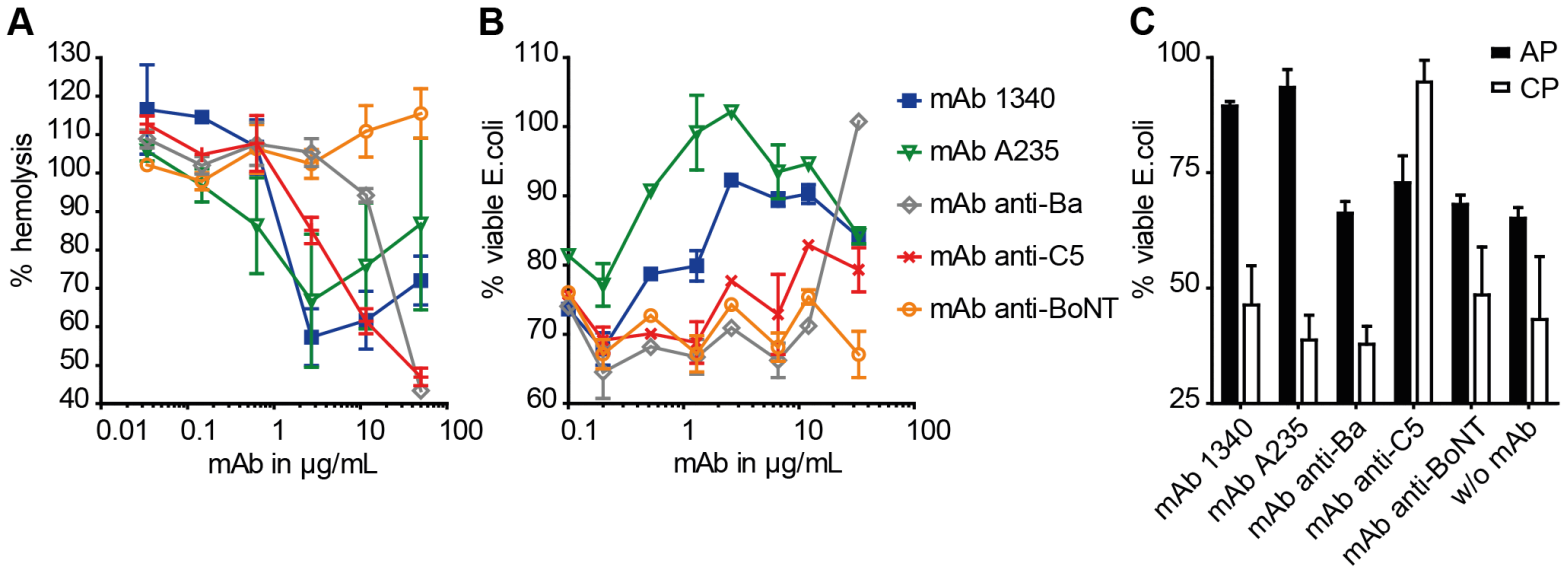
MAb 1340 inhibits function of the alternative complement system. (**A**) Effect of mAb 1340 on the alternative complement system was tested in a hemolysis assay. Human normal serum was preincubated with different antibodies (0.03–50 µg/mL). Lysis of sheep erythrocytes was measured at 414 nm after addition of the serum-mAb mixtures. Lysis of the erythrocytes was reduced after adding increasing concentrations of mAb anti-properdin (mAb 1340 (blue), mAb A235 (green)). Other complement system specific antibodies resulted in an inhibition of the complement system at higher concentrations (mAb anti-Ba (grey), mAb anti-C5 (red)). We observed lysis of sheep erythrocytes after incubation with a non-specific mAb anti-botulinum neurotoxin (BoNT, orange, isotype control). (**B**) Inhibition of NHS-associated *E. coli* lysis by mAb was tested. *E. coli* were cultivated in media with EGTA containing NHS and mAb in different concentrations (0.1–33 µg/mL). Percentages of viable *E. coli* cells were calculated in comparison to an untreated control. The *E. coli* lysis assay showed comparable inhibitory results with the hemolysis assay. MAb A235 showed a decrease in viable *E. coli* at concentrations above 1 µg/mL. (**C**) Blocking activity of different anti-complement mAb (6.6 µg/mL, anti-BoNT as isotype control) for EGTA containing NHS (AP alternative pathway, black columns) and NHS (CP classical pathway, white columns) mediated lysis of *E. coli* was analyzed. Anti-properdin mAb (mAb 1340, mAb A235) inhibited the alternative pathway but not the classical pathway. MAb anti-C5 blocked the lysis of *E. coli* mediated by alternative and classical pathway. For all figures means out of two independent experiments (n = 4±s.e.m., except for 4B concentrations 0.08, 0.52, 2.6, 12.1 µg/mL n = 2±s.e.m.) are shown.

Alorco et al. described the properdin monomer connecting vertexes as stabilization partners for the C3-convertase [16]. Binding of mAb 1340 to properdin showed an inhibition of the C3-convertase in functional complement assays (**Figure 4**). Human serum either preincubated with mAb 1340 or mAb A235 resulted from 1–10 µg/mL mAb in a reduced lysis of sheep red blood cells or *E. coli* cells, respectively (**Figure 4A, B**). Activity of mAb 1340 and mAb A235 resulted in a blocking peak at 1.3–2.7 µg/mL. At higher mAb concentrations cell lysis increased again likely due to complement reactivation via immune complex formation [56]. MAb 1340 and mAb A233 inhibited the binding of properdin to immobilized C3b and as a consequence thereof factor B binding was also diminished. MAb 235 did not interfere with properdin deposition on C3b coated plates but inhibited factor B binding to C3b (**Figure S3**).

The properdin targeting mAb 1340 and mAb A235 suppressed the alternative complement system more effectively, than anti-C5 or anti-Ba antibodies (**Figure 4A, B**). Five to fifteen times more antibody of C5 or Ba targeting mAbs were used to observe a comparable maximum inhibition of the lytic effect of human serum. An unspecific isotype control against botulinum toxin did not inactivate the complement pathways.

The function of properdin *in vivo* offers an opportunity for a selective manipulation of the alternative complement pathway. We tested the inhibition of classical and alternative pathway mediated by different mAb (**Figure 4C**). The alternative pathway was influenced by anti-properdin mAb and anti-C5 mAb and the classical complement system was only blocked by anti-C5 mAb. These results demonstrated that mAb 1340 inhibited the activity of properdin exclusively in the alternative complement pathway.

### Detailed structure of mAb 1340 as a basis for humanization

**Figure 5:**
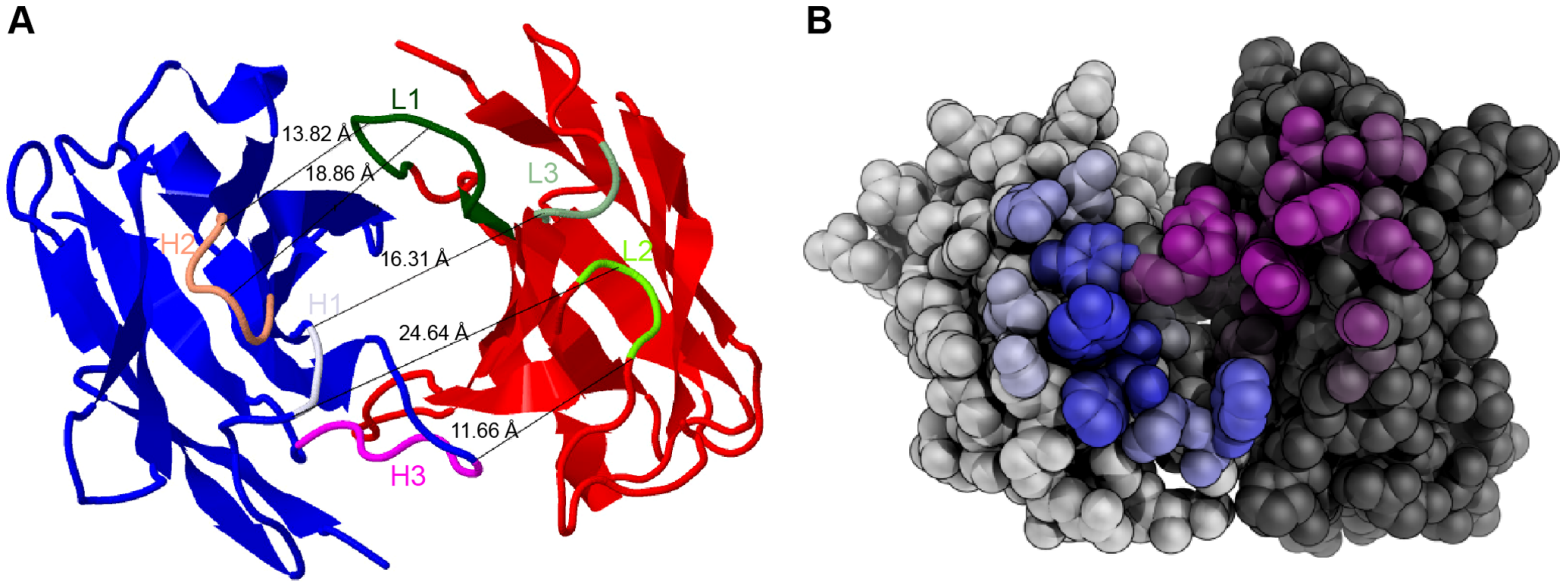
Structure of mAb 1340. (**A**) A model of mAb 1340 secondary structure shows the heavy (blue) and light (red) variable chain. The binding cleft is determined by six CDR loops (marked are overlapping amino acids from **Table 1**) either in the heavy chain H1 (orchid), H2 (salmon), H3 (magenta) or in the light chain L1 (dark green), L2 (chartreuse), L3 (springgreen). The distance between the chains varies between 11.66–24.64 Å. Modeling was performed with Rosetta. (**B**) Spherical display of mAb 1340 light (light grey) and heavy (dark grey) variable chain. The predicted amino acids for antigen contact are highlighted. The coloring of the residues is according to their contact probability value (the higher the probability the deeper the color). Modeling was performed with proABC.

MAb 1340 is a highly affine, specific and blocking antibody against properdin, with a mouse IgG 1 isotype and a κ-light chain. The immunoglobulin consisted of a highly glycosylated 35 kDa light chain and a 55 kDa heavy chain in SDS-PAGE (data not shown). Additionally, the amino acid sequence of the variable domain of light and heavy chain showed a very low humanness with a Z-score of −1.6 and −1.8, respectively. These structural properties suggested a prospective humanization or chimerization for an application *in vivo*. Therefore, the sequences of the hypervariable loops of the antigen binding region were analyzed. Seven different algorithms were used to predict the CDR (**Table 1**). All programs identified regions within the variable domains with overlapping amino acids (bold, underlined in **Table 1**). CDR-H2, CDR-H3 and CDR-L3 showed the highest consistency. Based on the amino acid sequence of the variable domains a three-dimensional model of mAb 1340 Fab was generated (**Figure 5A**). The framework was built from β-sheets and the CDRs formed α-helices. Predicted CDRs (bold, underlined in **Table 1**) formed an antigen binding cleft of 11.66–24.64 Å. The antibody binding groove was surrounded by amino acids residuals, which were predicted as contact amino acids with the antigen (**Figure 5B**). The defined analyses of mAb 1340 structure provided valuable information for further antibody modulations.

**Table 1:** Single letter amino acid sequence of complementarity-determining regions (CDR) of mAb 1340.

Algorithm/CDR	H1	H2	H3
Kabat	**SG**YWN	I**GYSGS**TFYNPSLKR	**GDDLFP**Y
Chothia	GDSIT**SG**	**GYSGS**	**GDDLFP**Y
IMGT	GDSIT**SG**YWN	**GYSGS**TF	TR**GDDLFP**Y
AbM	GDSIT**SG**YWN	FI**GYSGS**TF	**GDDLFP**Y
Contact	**SG**YWN	YMGFI**GYSGS**TF	TR**GDDLFP**
V-BASE	GDSIT**SG**Y	I**GYSGS**T	TR**GDDLFP**Y
Rosetta	GDSIT**SG**YWN	FI**GYSGS**TFYNPSLKR	**GDDLFP**Y

Algorithm/CDR	L1	L2	L3
Kabat	RASQNI**SDY**LQ	**YAS**QSIS	**QNGHSFPY**T
Chothia	RASQNI**SDY**LQ	**YAS**QSIS	**QNGHSFPY**T
IMGT	QNI**SDY**	**YAS**	**QNGHSFPY**T
AbM	RASQNI**SDY**LQ	**YAS**QSIS	**QNGHSFPY**T
Contact	**SDY**LQWY	LLIK**YAS**QSI	**QNGHSFPY**
V-BASE	QNI**SDY**	**YAS**	**QNGHSFPY**T
Rosetta	RASQNI**SDY**LQ	**YAS**QSIS	**QNGHSFPY**T

H variable domain of heavy chain, L variable domain of light chain; Kabat, Cothia, IMGT, AbM, Contact, VBASE2 and Rosetta are different CDR prediction algorithms; bold and underlined marked amino acids are similar in all sequence.

### MAb 1340 detects specifically and reproducibly properdin from human blood in a sandwich ELISA

**Figure 6:**
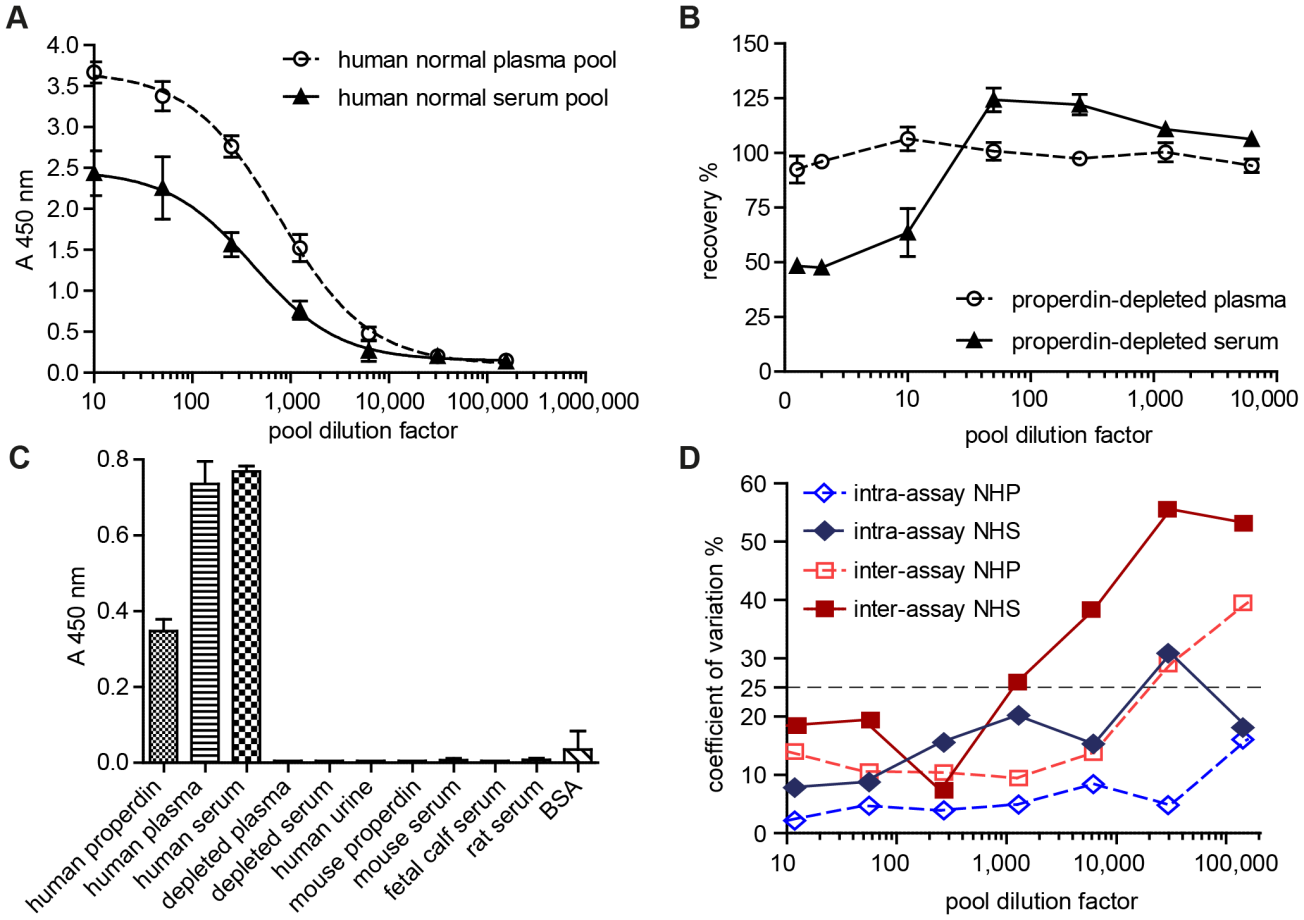
A sandwich ELISA detected human properdin from blood samples highly sensitively, specifically and reproducibly. (**A**) Serial dilutions of human normal plasma and serum pools (1∶10–1∶156,250) were used as a reference curve for properdin detection from human samples in a sandwich ELISA. Detection of unknown samples was reliable in a plasma and serum dilution range from 1∶100 to 1∶1,000. The EC50 of the plasma curve was at 1∶800 and for serum at 1∶400, respectively. The mean value of three independent measurements with duplicates is shown. The reference curves were representable for all performed experiments. (**B**) Recovery rates of 100 ng/mL properdin from properdin-depleted NHP and NHS are shown. Plasma matrix did not interfere with properdin recovery. Serum dilution 1∶50 and higher gives a recovery rate between 75–125%. (**C**) The described sandwich ELISA specifically detected human properdin only either purified or from blood samples. Properdin from mouse, rat or calf serum showed no signal in an ELISA with immobilized antigens. Other negative controls such as human blood depleted from properdin, human normal urine or bovine serum albumin were not detected. Two independent experiments with duplicates were performed. (**D**) Intra-assay and inter-assay coefficient of variation for different plasma and serum concentrations in the sandwich ELISA displayed high reproducibility. Serial dilutions of a human normal plasma and serum pool (1∶10–1∶156,250) were determined. The sandwich ELISA shows CV values below 25% for plasma dilutions from 1∶10 to 1∶10,000 and for serum dilutions 1∶10 to 1∶1,000, respectively.

To determine the properdin concentration in different patient cohorts a sandwich-ELISA based on mAb 1340 was developed and validated for human blood samples (**Figure 6**). Properdin detection from human plasma was more sensitive than from human serum with EC50 values of 30 ng/mL (1∶800) and 50 ng/mL (1∶400), respectively. The linear range of the plasma standard curve covered dilutions from 1∶200 to 1∶2,000 and the serum standard curve showed a linear range from 1∶100 to 1∶1,000 (**Figure 6A**). Recovery rate of 100 ng/mL properdin spiked into properdin depleted human plasma or serum differed over the tested sample dilution range. Acceptable recovery rates between 80–125% were obtained for all tested plasma concentrations (**Figure 6B**). Plasma did not alter the detection of spiked properdin. In contrast, serum had to be diluted at least 1∶50 to avoid a masking matrix effect (**Figure 6B**) [57]. We tested the specificity of the ELISA for different animal sera and only human properdin was detected. There were no false positive results for mouse, rat or bovine properdin (**Figure 6C**). Additionally, the assay showed a high reproducibility for plasma dilutions less than 1∶10,000 with a CV below 25% (**Figure 6D**). For serum samples a difference in within-plate and plate-to-plate consistency was determined. The intra-assay reproducibility (CV<25% at 1∶10,000) for serum was better than the inter-assay reproducibility (CV<25% at 1∶1,000) (**Figure 6D**). The analyses of serum and plasma samples within the linear range of the assay were reproducible. We chose a dilution of 1∶700 for all unknown plasma and serum samples. This dilution is in the linear range of the assay, gives acceptable recovery and sufficient reproducibility.

### Properdin concentrations in patients with age-related macular degeneration or rheumatic diseases and control sera are similar

**Figure 7:**
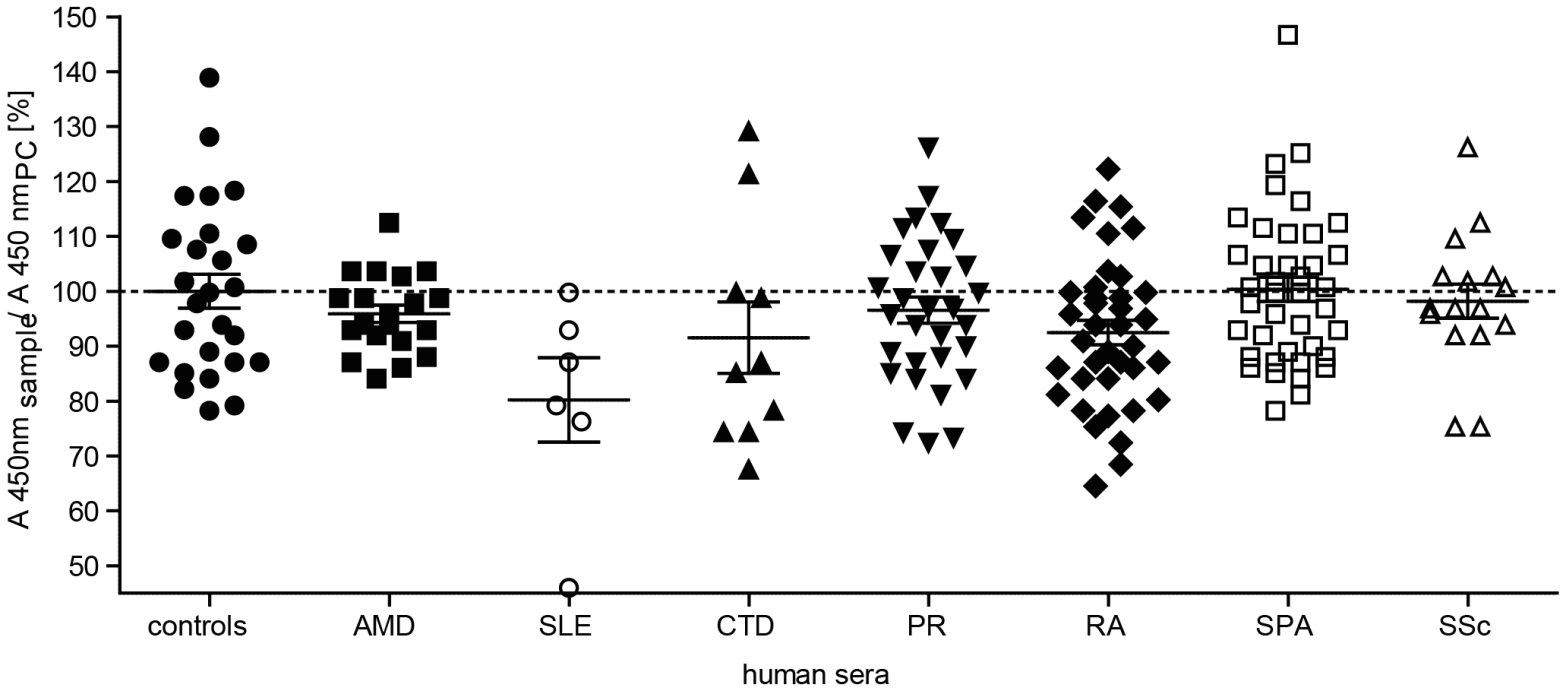
Systemic properdin concentrations in patient serum compared to healthy controls. Serum samples of healthy blood donors (controls, n = 26), patients with age-related macular degeneration (AMD, n = 20), systemic lupus erythematosus (SLE, n = 6), connective tissue diseases (CTD, n = 10), polymyalgia rheumatica (PR, n = 31), rheumatoid arthritis (RA, n = 38), spondyloarthritis (SPA, n = 40) and systemic sclerosis (SSc, n = 16) were diluted in PBS (1∶700). Properdin amount in serum samples was compared to a positive control (PC, NHS pool 1∶700, ratio on y-axis) using the described ELISA for human properdin (see **Figure 6**). Patient and control groups showed a properdin amount in serum between 66–155% of the positive control. This corresponded to 13–30 µg/mL properdin. There was no significant difference in properdin concentration between the cohorts (two-tailed, paired t-test, P>0.001).

Complement activation is involved in AMD and different rheumatoid diseases [58]. The properdin amount of AMD patients (n = 20, mean 98% of positive control) corresponded to the concentration in the healthy control group (n = 26, mean 102% of the positive control) (**Figure 7**). There was also no significant difference in the ELISA signal for properdin detection in the group of rheumatoid diseases versus the control cohort (**Figure 7**). Patients with connective tissue diseases (CTD, n = 10), polymyalgia rheumatica (PR, n = 31), rheumatoid arthritis (RA, n = 38), spondyloarthritis (SPA, n = 40) and systemic sclerosis (SSc, n = 16) showed no discriminable properdin detection compared to the positive control (means were 94%, 99%, 95%, 103% and 100%, respectively). Patients with systemic lupus erythematosus (SLE, n = 6) showed a lower but not significant signal with 82% of the control (two-tailed, paired t-test, P = 0.2496). These results indicate a consistent systemic properdin serum concentration for the analyzed diseases.

## Discussion

### MAb 1340 as a future complement therapeutic?

Complement protein deficiency or partial dysfunction can lead to a disturbed homeostasis of the alternative pathway and as a consequence result in pathological inflammatory processes. Diseases such as AMD, rheumatic diseases and aHUS have been associated with uncontrolled alternative complement activation [58]. Different therapeutic interventions are under development to specifically target the alternative pathway although this may be a challenging task at hand [33]. There is a difficult balancing act to inhibit the alternative complement pathway and at the same time maintain the physiological function of this important player of the innate immune system.

Therapeutic compounds like Eculizumab (anti-C5 mAb) or Compstatin (anti-C3 peptide) target the central serine-proteases of all three complement pathways and are tested in clinical studies for aHUS as well as AMD, respectively [59], [60]. Developments like CFH supplements or anti-properdin antibodies offer the opportunity to influence regulators of the alternative complement system, while the proteases are only indirectly blocked [61], [62]. Beneficial effects of properdin inhibition in mouse models for complement-mediated tissue injuries like arthritis or abdominal aortic aneurysm have been shown [26]–[29], [63]. The novel mAb 1340 described here is a mouse antibody with high affinity and inhibiting activity against human native properdin. The inhibitory efficiency of mAb 1340 is comparable to previously described anti-human properdin immunoglobulins [37], [64]. Complement-targeting antibodies with a specificity for C5 and factor B required higher immunoglobulin concentrations to block the complement pathway [65], [66]. Given that the concentration of properdin in human serum is 4–25 µg/mL, these data suggested that a dose of 3–13 µg/mL of mAb 1340 would be sufficient to block alternative complement activation in a systemic application [11]. This is in the range of established bioavailability of approved therapeutics (Adalimumab 3.1–6.3 µg/mL, Alemtuzumab 2.5–6.1 µg/mL) [67], [68].

Bansal-Gupta et al. described a properdin targeting mAb, which also showed blocking activity [37]. This antibody inhibited the binding of C3 to properdin and interfered with C3-convertases of all three complement pathways. Importantly, the reported mAb 1340 inhibits exclusively the alternative pathway by influencing the interaction of C3 with properdin. MAb 1340 precipitated properdin in association with high amounts of C3(H_2_O), human IgG1 and clusterin from human serum. These results suggest that properdin circulates in a protein complex in human blood and is acting in concert with C3(H_2_O) or C3b as an initiator of complement activation [44], [69]. Previous studies have demonstrated that properdin binds Ba, the N-terminal part of factor B and the von Willebrand factor type-A domain of the c-terminal cleavage product Bb [16], [17]. We have not observed factor B fragments in coimmunoprecipitation and combined mass spectrometry analysis. The exact epitope of mAb 1340 is so far unknown. We hypothesize that mAb 1340 binds to the vertexes of properdin oligomers and inhibits binding of C3b and Factor B.

The activation of the C3-convertase can further be influenced by unknown protective mechanisms which inhibit properdin-C3(H_2_O)/C3b binding to cell surfaces [10]. An inhibitory effect could be provided by clusterin, which was also identified in our study from serum in combination with mAb 1340-properdin-C3(H_2_O)/C3b. Clusterin suppresses cytolytic activity of terminal components of the complement pathway C5b–7 [70]. While under oxidative stress or accumulation of non-native deposits, clusterin binds to stress epitopes and prevents proteins from aggregation [71], [72]. After clusterin dissociates from the C3b-properdin or C5b–7, the complexes can bind to surfaces.

Previously established properdin blocking mAbs tested in our study were commercially available mouse antibodies, but nothing is known about the amino acid sequences or the structure of the antigen-binding sites. However, this knowledge is crucial for future application in humans. Therapeutic antibodies in clinical studies are mainly humanized or chimeric antibodies [73]. MAb 1340 is an antibody with a low humanness score, that could elicit a human anti-mouse antibody response, fix complement at the Fc-region and therefore result in a systemic inflammatory response [74]. Cloning of mAb 1340 CDRs into a human immunoglobulin framework is required and would result in reduced immunogenicity. The described three-dimensional model and detailed CDR analysis of mAb 1340 helps to preserve the antibody specificity of mAb 1340 for human oligomeric properdin after CDR-grafting, back mutations and affinity maturation, required for future therapeutic applications [75]. MAb 1340 allows for a pathway specific inhibition and has a high potential for amelioration of inflammation *in vivo*.

### Determination of stable systemic properdin concentrations

The complement system and complement diagnostics were described more than a century ago. However, complement analysis in the clinic is limited to C3, C4, C1-inhibitor and overall complement activity in hemolytic assays [76]. Detection of properdin deficiency is a challenge as alternative pathway hemolytic activity is low but often within the normal range [77]. The techniques improved in last years, but still lack standardization, feasibility for multiparameter and bulk analyses [76], [78]. Properdin diagnostics are done by radial immunodiffusion, complement fixation assay or ELISA [32], [34], [57], [79]. A described sandwich ELISA used polyclonal antibodies and detected properdin from serum samples [57]. This ELISA showed similar recovery rates and day-to-day variance as the reported ELISA with mAb 1340. Hoffmann et al. described a high dose hook effect in the ELISA [57]. Our ELISA showed a dose-dependent slope of the standard curve for serum and plasma without any inhibited properdin detection at higher blood concentrations. The novel properdin sandwich ELISA-based detection technique offers an opportunity for a precise, reproducible and routine measurement of properdin concentration in serum and plasma samples.

To our knowledge there is no standardized screening in complement diagnostics for properdin deficiency or altered blood titers in diseases so far. Therefore, effects of properdin deficiency could easily be underdiagnosed. Properdin deficiencies are orphan diseases, which are associated with increased susceptibility to meningococcal disease [80]. There is also evidence that systemic and local properdin concentrations are modified in certain immune-mediated disorders. Systemically decreased properdin concentrations were reported for autoantibody-associated diseases like neuromyelitis optica, Schönlein-Henoch syndrome and SLE [32]–[34]. Although not significant with the present sample size of patients, we reproduced a reduction in systemic properdin amounts in SLE patients's serum. For other rheumatic diseases and AMD a correlation with complement activation was described [81], [82]. However, we could not find any systemic changes in the properdin concentrations in patients with AMD, connective tissue diseases, polymyalgia rheumatica and spondyloarthritis. Concordant with existing studies, we also did not observe any significant deviation of serum titers for autoantibody-associated diseases like rheumatoid arthritis or systemic sclerosis [31], [83]. Prompted by previous reports about properdin reduction in synovial fluid in rheumatoid arthritis and local properdin depletion in AMD affected eyes, we suggest a local but not systemic change of properdin concentrations for AMD and rheumatoid arthritis [31], [84]. Animal experiments showed local properdin-dependent cell-lysis can be caused by systemically circulating properdin [28]. Thus, therapeutically targeting systemic properdin and therefore ameliorating the alternative complement system with mAb 1340 could be effective and feasible in locally complement-mediated diseases like arthritis or abdominal aortic aneurysm [25].

## Conclusions

The recent success of complement inhibitors and reports about properdin-associated diseases, illustrate a promising potential for anti-properdin treatment in complement-mediated tissue injuries. We anticipate that the development of the novel distinct properdin inhibiting mAb 1340 and our specific properdin detection system will be instrumental to further characterization of properdin and its impact on human pathologies.

## Supporting Information

Figure S1 **MAb 1340 interacts with different thrombospondin like repeats of properdin.** Properdin is a 55 kDa protein and consists of seven thrombospondin like repeats (TSR 0 yellow, TSR 1 purple, TSR 2 red, TSR 3 grey, TSR 4 green, TSR 5 blue, TSR 6 cyan). Each TSR is built of 49–84 amino acids with connecting amino acids (yellow) between different TSRs. The short C-terminus is depicted in grey. (**A, B, C**) *In silico* modeling of docking showed binding of mAb 1340 heavy (blue) and light (red) variable domain to different TSRs of a properdin monomer (PDB 1W0S, A chain). The different CDRs based on identical sequences of **Table 1** are shown. The large picture shows the interaction of properdin and mAb 1340. The inlay enlarges the binding regions between CDRs and TSR. (**A**) Prediction algorithm of PatchDock showed an interaction of the CDRs H1, H2, L1 and L2 with TSR 1. (**B**) Binding of CDR H3, L1, L2 to TSR 3 and H2 to CDR 4 were described by the HexServer algorithm. (**C**) A third algorithm docked CDR H2, L1 and L3 to TSR 4. Docking was performed with GRAMM-X server. (**D**) A proposed model for a native properdin multimer is shown [16]. Two properdin monomers form a loop of four TSRs at each connecting point, respectively. In this model mAb 1340 (grey circle) interacts with the connecting points, based on the three *in silico* docking algorithms.
(TIF)

Figure S2 **Determination of properdin thrombospondin like repeat specificity of mAb 1340 shows only binding to full length properdin.** (**A**) Human properdin and TSR subunits conjugated to maltose binding protein (MBP, 500 ng) were separated on a 15% SDS-Gel and transferred on a PVDF membrane. Protein detection was performed with mAb 1340. A positive binding was reportable for full length human properdin but not for the different TSR subunits. (**B**) A competitive ELISA for mAb 1340 binding to human properdin or TSR was performed. Different concentrations of mAb 1340 (0.06–0.012 µg/mL, bars) were preincubated with different antigens in solution (100 µg/mL, depicted on the x-axis). Antibody/antigen mixtures were added to an ELISA plate, which was coated with human properdin. MAb 1340 binding to immobilized properdin was detected. Soluble human properdin inhibited the binding of mAb 1340 to immobilized properdin. None of the TSRs did inhibit the binding of different concentrations of mAb 1340 to immobilized properdin. Shown is an example of two independent experiments with similar results.
(TIF)

Figure S3 **MAb 1340 inhibits properdin and factor B binding to C3b.** The complement blocking activity of mAb 1340 (blue), mAb A233 (purple), mAb A235 (green) and mAb anti-BoNT (orange) were tested on C3b coated plates. MAbs were serially diluted (0.01–10 µg/mL) in (**A**) 10% or (**B**) 20% NHS/MgEGTA buffer and incubated on a blocked C3b plate. (**A**) Properdin deposition was detected with goat anti-properdin pAb and (**B**) complement factor B (CFB) deposition was analyzed with goat anti-CFB pAb. Signal was determined using a peroxidase conjugated anti-goat antibody, TMB and measurement at 450 nm. All data were normalized to the NHS measurements without mAb (set to 100%). Shown are means ((**A**) n = 6±s.e.m., (**B**) n = 3±s.e.m.) out of three independent experiments. MAb 1340 and mAb A233 blocked properdin and CFB deposition on immobilized C3b. MAb A235 inhibited not properdin deposition but CFB detection on C3b coated plates. The unspecific isotype control did inhibit the complement activity.
(TIF)

Checklist S1 **The ARRIVE Guidelines Checklist.**
(DOCX)

Material and Methods S1 **Competetive ELISA, Inhibition of complement deposition, Software.**
(DOCX)
