# Supplementary figures and images for: A Novel Antibody against Human Properdin Inhibits the Alternative Complement System and Specifically Detects Properdin from Blood Samples
Source: PLoS One. 2014 May 5;9(5):e96371. doi: 10.1371/journal.pone.0096371 (PMC4010523; doi:10.1371/journal.pone.0096371)

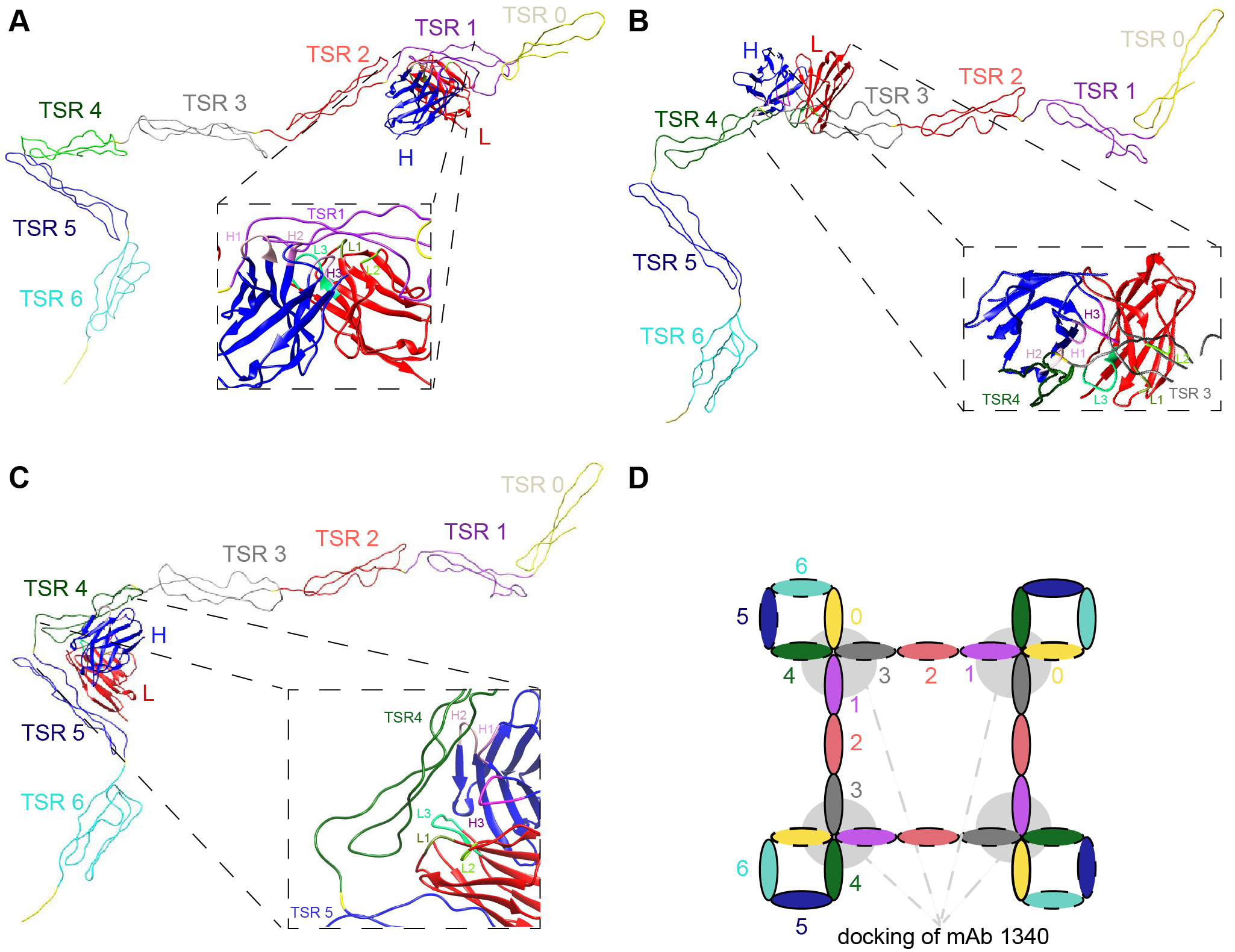

Supplement: Figure S1 — MAb 1340 interacts with different thrombospondin like repeats of properdin. Properdin is a 55 kDa protein and consists of seven thrombospondin like repeats (TSR 0 yellow, TSR 1 purple, TSR 2 red, TSR 3 grey, TSR 4 green, TSR 5 blue, TSR 6 cyan). Each TSR is built of 49–84 amino acids with connecting amino acids (yellow) between different TSRs. The short C-terminus is depicted in grey. (A, B, C) In silico modeling of docking showed binding of mAb 1340 heavy (blue) and light (red) variable domain to different TSRs of a properdin monomer (PDB 1W0S, A chain). The different CDRs based on identical sequences of Table 1 are shown. The large picture shows the interaction of properdin and mAb 1340. The inlay enlarges the binding regions between CDRs and TSR. (A) Prediction algorithm of PatchDock showed an interaction of the CDRs H1, H2, L1 and L2 with TSR 1. (B) Binding of CDR H3, L1, L2 to TSR 3 and H2 to CDR 4 were described by the HexServer algorithm. (C) A third algorithm docked CDR H2, L1 and L3 to TSR 4. Docking was performed with GRAMM-X server. (D) A proposed model for a native properdin multimer is shown [16]. Two properdin monomers form a loop of four TSRs at each connecting point, respectively. In this model mAb 1340 (grey circle) interacts with the connecting points, based on the three in silico docking algorithms. (TIF) [file pone.0096371.s001.tif]

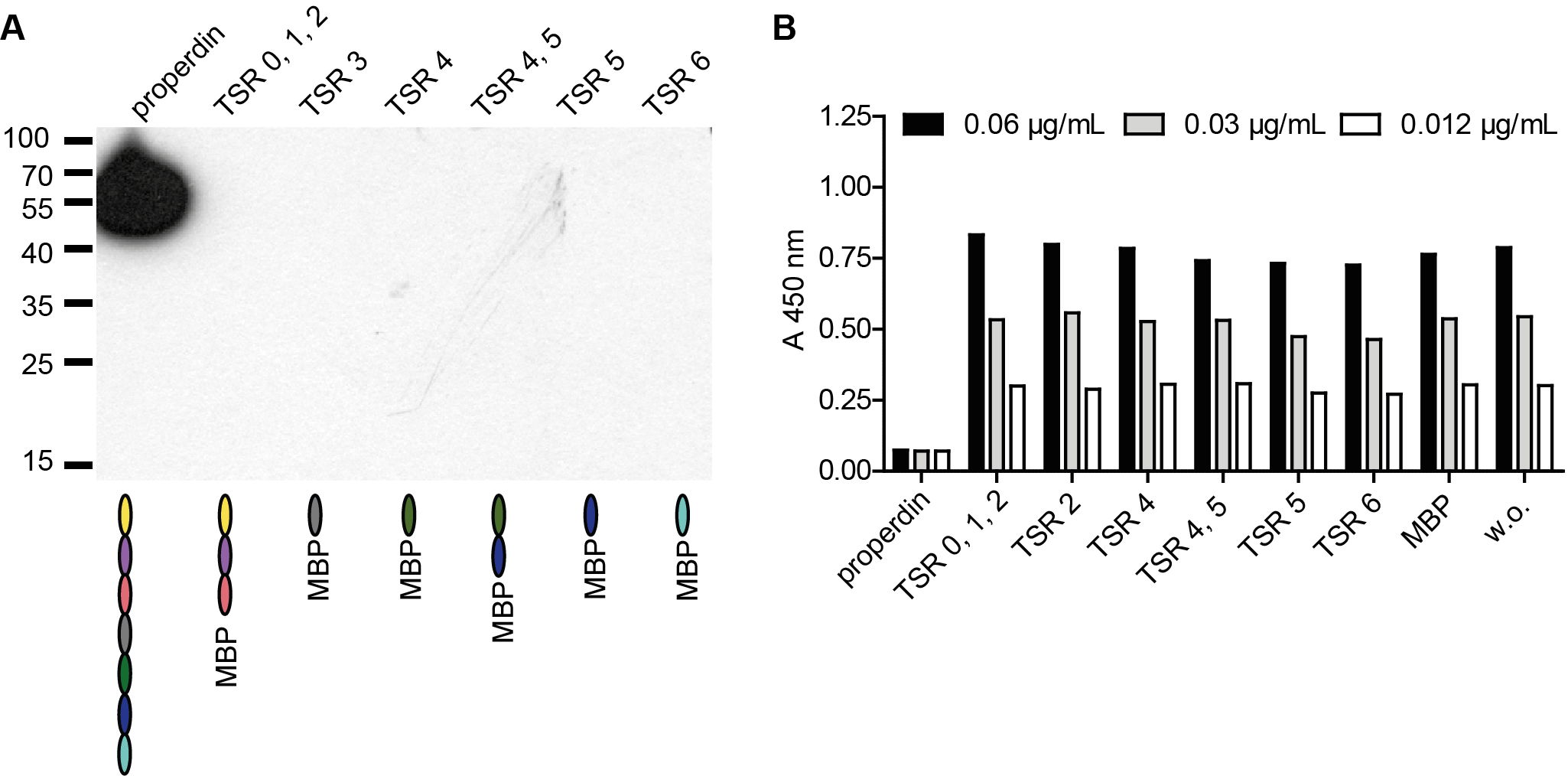

Supplement: Figure S2 — Determination of properdin thrombospondin like repeat specificity of mAb 1340 shows only binding to full length properdin. (A) Human properdin and TSR subunits conjugated to maltose binding protein (MBP, 500 ng) were separated on a 15% SDS-Gel and transferred on a PVDF membrane. Protein detection was performed with mAb 1340. A positive binding was reportable for full length human properdin but not for the different TSR subunits. (B) A competitive ELISA for mAb 1340 binding to human properdin or TSR was performed. Different concentrations of mAb 1340 (0.06–0.012 µg/mL, bars) were preincubated with different antigens in solution (100 µg/mL, depicted on the x-axis). Antibody/antigen mixtures were added to an ELISA plate, which was coated with human properdin. MAb 1340 binding to immobilized properdin was detected. Soluble human properdin inhibited the binding of mAb 1340 to immobilized properdin. None of the TSRs did inhibit the binding of different concentrations of mAb 1340 to immobilized properdin. Shown is an example of two independent experiments with similar results. (TIF) [file pone.0096371.s002.tif]

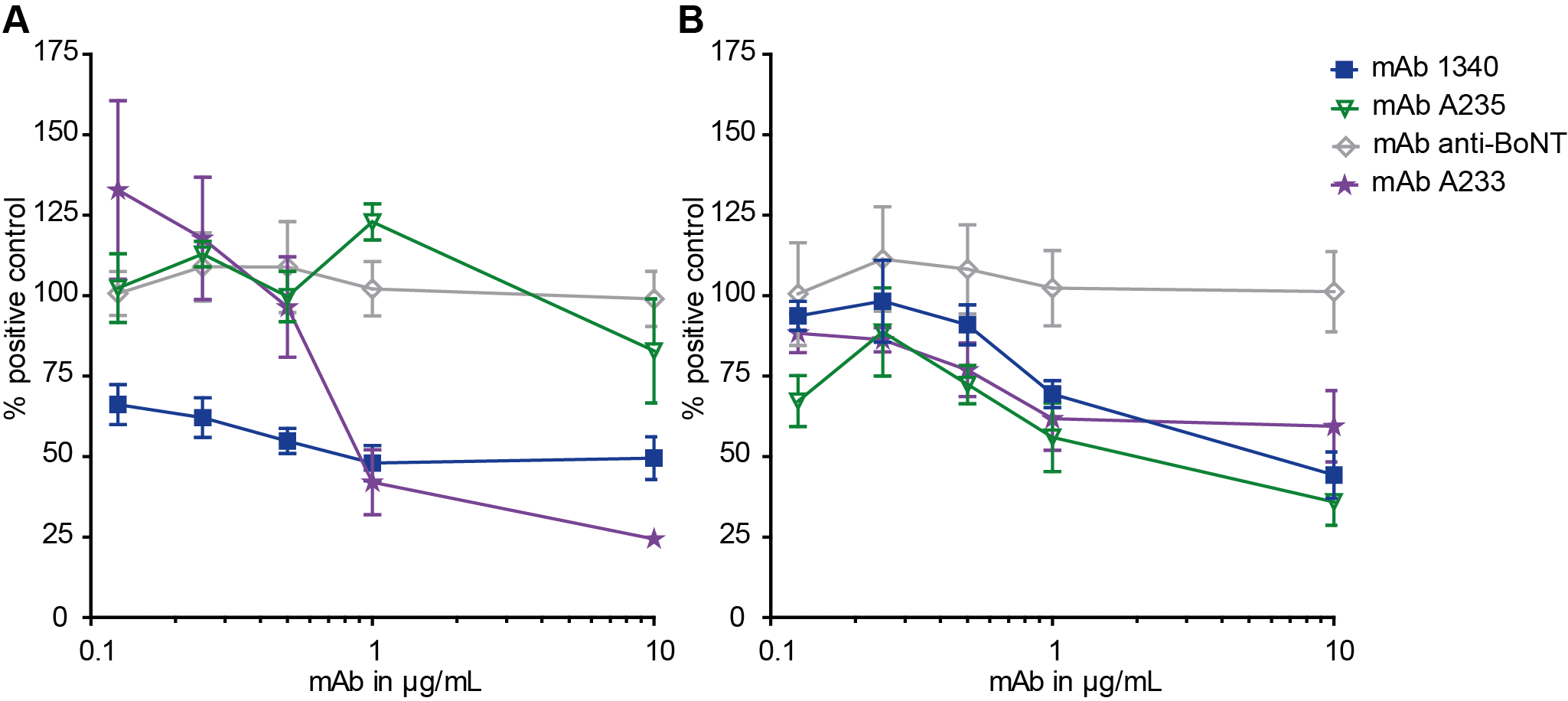

Supplement: Figure S3 — MAb 1340 inhibits properdin and factor B binding to C3b. The complement blocking activity of mAb 1340 (blue), mAb A233 (purple), mAb A235 (green) and mAb anti-BoNT (orange) were tested on C3b coated plates. MAbs were serially diluted (0.01–10 µg/mL) in (A) 10% or (B) 20% NHS/MgEGTA buffer and incubated on a blocked C3b plate. (A) Properdin deposition was detected with goat anti-properdin pAb and (B) complement factor B (CFB) deposition was analyzed with goat anti-CFB pAb. Signal was determined using a peroxidase conjugated anti-goat antibody, TMB and measurement at 450 nm. All data were normalized to the NHS measurements without mAb (set to 100%). Shown are means ((A) n = 6±s.e.m., (B) n = 3±s.e.m.) out of three independent experiments. MAb 1340 and mAb A233 blocked properdin and CFB deposition on immobilized C3b. MAb A235 inhibited not properdin deposition but CFB detection on C3b coated plates. The unspecific isotype control did inhibit the complement activity. (TIF) [file pone.0096371.s003.tif]
