# Supplementary material for: A Novel Antibody against Human Properdin Inhibits the Alternative Complement System and Specifically Detects Properdin from Blood Samples
Source: PLoS One. 2014 May 5;9(5):e96371. doi: 10.1371/journal.pone.0096371 (PMC4010523; doi:10.1371/journal.pone.0096371)
Supplement: Checklist S1 — The ARRIVE Guidelines Checklist. (DOCX) [file pone.0096371.s004.docx]

The ARRIVE Guidelines Checklist

|  | ITEM | Statement | Section |
| --- | --- | --- | --- |
| Title | 1 | A Novel Antibody against Human Properdin Inhibits the Alternative Complement System and Specifically Detects Properdin from Blood Samples | Title |
| Abstract | 2 | The complement system is an essential part of the innate immune system by acting as a first line of defense which is stabilized by properdin, the sole known positive regulator of the alternative complement pathway. Dysregulation of complement can promote a diversity of human inflammatory diseases which are treated by complement inhibitors. Here, we generated a novel blocking monoclonal antibody (mAb) in Balb/c mice against properdin and devised a new diagnostic assay for this important complement regulator. Mouse mAb 1340 specifically detected native properdin from human samples with high avidity. MAb 1340 inhibited specifically the alternative complement mediated cell lysis within a concentration range of 0.5–2.7 µg/mL. Thus, in vitro anti-properdin mAb 1340 was fifteen times more efficient in blocking the complement system as compared to anti-C5 or anti-Ba antibodies. Computer-assisted modelling suggested a three-dimensional binding epitope in a properdin-C3(H_2_O)-clusterin complex to be responsible for the inhibition.  Recovery of properdin in a newly established sandwich ELISA using mAb 1340 was determined at 80–125% for blood sample dilutions above 1:50. Reproducibility assays showed a variation below 25% at dilutions less than 1:1,000. Systemic properdin concentrations of healthy controls and patients with age-related macular degeneration or rheumatic diseases were all in the range of 13–30 µg/mL and did not reveal significant differences.  These initial results encourage further investigation into the functional role of properdin in the development, progression and treatment of diseases related to the alternative complement pathway. Thus, mAb 1340 represents a potent properdin inhibitor suitable for further research to understand the exact mechanisms how properdin activates the complement C3-convertase and to determine quantitative levels of properdin in biological samples. | Abstract |
| Background | 3 | Therapeutic compounds like Eculizumab (anti-C5 mAb) or Compstatin (anti-C3 peptide) target the central serine-proteases of all three complement pathways and showed effectiveness in clinical studies for aHUS as well as AMD, respectively [56,57]. Developments like CFH supplements or anti-properdin antibodies offer the opportunity to influence regulators of the alternative complement system, while the proteases are only indirectly blocked [58,59]. Beneficial effects of properdin inhibition in mouse models for complement-mediated tissue injuries like arthritis or abdominal aortic aneurysm have been shown [26–29,60]. The novel mAb 1340 described here is a mouse antibody with high affinity and inhibiting activity against human native properdin. | Whole Paper |
| Objectives | 4 | Immunization of Balb/c mice results in specific IgG generation. Spleen cells are used for the generation of monoclonal antibodies. | Results |
| Ethical statement | 5 | All mice were handled according to the good animal practice in science. This study was carried out in strict accordance with the recommendations in the Guidelines for the Care and Use of Laboratory Animals of the Federation of European Laboratory Animal Science Associations. The protocol was approved by the Committee on the Ethics of Animal Experiments of the the regional agency for animal health Regierung der Oberpfalz, Veterinärwesen (permit number: TV 54-2532.4-04/12). All immunizations and bleedings were performed under inhalational anesthesia (Isoflurane), and all efforts were made to minimize suffering. | Methods |
| Study design | 6 | Two mice were immunized. No other study design was performed. | Methods |
| Experimental procedures | 7 | Mouse monoclonal antibodies against human properdin were generated as previously described [39]. Briefly, six weeks-old female Balb/c mice (Charles River Laboratories, Sulzfeld, Germany) were immunized five times with 25 µg human, purified properdin (Quidel, San Diego, USA). Antigens were applied subcutaneously in complete Freund`s adjuvants for priming immunization and incomplete Freund`s adjuvants for follow-up immunizations. | Methods |
| Experimental animals | 8 | six weeks-old female Balb/c mice (Charles River Laboratories, Sulzfeld, Germany) | Methods |
| Housing and husbandry | 9 | Central animal facility University Regensburg, specific pathogen free housing | Methods |
| Sample size | 10 | Two mice were immunized. Smallest possible sample size was used. No other calculations were performed. | Methods |
| Allocating animals to experimental groups | 11 | N/A |  |
| Experimental outcomes | 12 | Both animals developed an antibody titer against human properdin. MAb 1340 was isolated and characterized. | Results |
| Statistical methods | 13 | N/A |  |
| Baseline data | 14 | Antibody serum titer before immunization showed no titer against human properdin. |  |
| Numbers analyzed | 15 | Two mice were immunized. Only one mouse was successfully fused with myeloma cells. |  |
| Outcomes and estimation | 16 | N/A |  |
| Adverse events | 17 | N/A |  |
| Interpretation/scientific implications | 18 | We anticipate that the development of the novel distinct properdin inhibiting mAb 1340 and our specific properdin detection system will be instrumental to further characterization of properdin and its impact on human pathologies. | Results/ Discussion |
| Generalizability/ translation | 19 | Antibodies are generated against foreign antigens. This could be used for vaccination strategies und for generation and isolation of antibodies as tools. |  |
| Funding | 20 | This work was supported by a grant from the PRO RETINA foundation (http://www.proretina.de, DP) and in part by grants from the German Research Foundation (DFG, http://www.dfg.de/) DFG Sk46/2-1 (CS)WE1259/18-1 (BHFW), WE1259/19-1 (BHFW), The Ruth and Milton Steinbach Foundation New York (BHFW) and the Alcon Research Institute (http://www.alcon.com/, BHFW).  The funders had no role in study design, data collection and analysis, decision to publish, or preparation of the manuscript. |  |
