# Supplementary material for: A Novel Antibody against Human Properdin Inhibits the Alternative Complement System and Specifically Detects Properdin from Blood Samples
Source: PLoS One. 2014 May 5;9(5):e96371. doi: 10.1371/journal.pone.0096371 (PMC4010523; doi:10.1371/journal.pone.0096371)
Supplement: Material and Methods S1 — Competetive ELISA, Inhibition of complement deposition, Software. (DOCX) [file pone.0096371.s005.docx]

**Materials and Methods S1**

**Material**

TSR subunits were a kindly provided by U. Kishore (Brunel University, Uxbridge, United Kingdom) [1]. C3b and pAb goat anti-properdin was obtained from Complement technologies (Tyler, USA). PAb goat anti-complement factor B and peroxidase conjugated donkey anti-goat was purchased from Calbiochem/ Merck (Darmstadt, Germany).

**Competitive ELISA**

MaxiSorp plates were coated with 1 µg/mL properdin in PBS (overnight, 4 °C). All incubation steps were completed with three subsequent washing steps with wash buffer (PBS, 0.1% Tween 20). MAb 1340 was serially diluted in 0.1% BSA/PBS (0.06–0.012 µg/mL) and preincubated with different antigens in solution (100 µg/mL, 30 min). After blocking with blocking buffer (PBS, 0.1% Tween 20, 2% skim milk) antibodies-antigen mixtures were added to the properdin coated plate (1 h). Detection of antibody binding to the solid phase was performed with a peroxidase conjugated anti-mouse IgG antibody and TMB. Signal was determined at 450 nm.

**Inhibition of complement deposition**

MaxiSorp plates were coated with 1 µg/mL C3b in PBS (overnight, 4 °C). All incubation steps were completed with three subsequent washing steps with wash buffer (TBS, 0.02% Tween 20). After blocking with blocking buffer (TBS, 0.02% Tween 20, 2% bovine serum albumin) either 10% (properdin detection) or 20% HNS (factor B detection) in MgEGTA buffer was spiked with serial diluted anti-complement or control antibodies (10–0.01 mg/mL) and were added to the plates (30 min, 37 °C). Detection was performed either with pAb goat anti-properdin (1:2500) or pAb goat anti-complement factor B (1:250) with subsequent peroxidase conjugated donkey anti-goat antibody (1:10.000, each 30 min, 37 °C). Binding of mAb was analyzed using a peroxidase conjugated anti-mouse antibody (30 min, 37 °C). Signals were determined after incubation with TMB at 450 nm. Data were normalized to properdin or factor B deposition of 20% untreated NHS, respectively.

**Software**

*In silico* docking of the variable region of mAb 1340 to properdin (PDB ID: 1W0S, A chain) was simulated with PatchDock, HexServer and GRAMM-X server [2–4].

**References S**

1. Perdikoulis M V, Kishore U, Reid KB (2001) Expression and characterisation of the thrombospondin type I repeats of human properdin. Biochim Biophys Acta 1548: 265–277. Available: http://www.ncbi.nlm.nih.gov/pubmed/11513971.

2. Schneidman-Duhovny D, Inbar Y, Nussinov R, Wolfson HJ (2005) PatchDock and SymmDock: servers for rigid and symmetric docking. Nucleic Acids Res 33: W363–7. Available: http://www.pubmedcentral.nih.gov/articlerender.fcgi?artid=1160241&tool=pmcentrez&rendertype=abstract. Accessed 31 October 2013.

3. Tovchigrechko A, Vakser I a (2006) GRAMM-X public web server for protein-protein docking. Nucleic Acids Res 34: W310–4. Available: http://www.pubmedcentral.nih.gov/articlerender.fcgi?artid=1538913&tool=pmcentrez&rendertype=abstract. Accessed 30 October 2013.

4. Macindoe G, Mavridis L, Venkatraman V, Devignes M-D, Ritchie DW (2010) HexServer: an FFT-based protein docking server powered by graphics processors. Nucleic Acids Res 38: W445–9. Available: http://www.pubmedcentral.nih.gov/articlerender.fcgi?artid=2896144&tool=pmcentrez&rendertype=abstract. Accessed 30 October 2013.
